# A comprehensive review on biomarkers associated with painful temporomandibular disorders

**DOI:** 10.1038/s41368-021-00129-1

**Published:** 2021-07-29

**Authors:** Mayank Shrivastava, Ricardo Battaglino, Liang Ye

**Affiliations:** 1 Department of Diagnostic and Biological Sciences, School of Dentistry, University of Minnesota, Minneapolis, MN USA; 2 Department of Rehabilitation Medicine, Medical School, University of Minnesota, Minneapolis, MN USA

**Keywords:** Biomarkers, Molecular biology

## Abstract

Pain of the orofacial region is the primary complaint for which patients seek treatment. Of all the orofacial pain conditions, one condition that possess a significant global health problem is temporomandibular disorder (TMD). Patients with TMD typically frequently complaints of pain as a symptom. TMD can occur due to complex interplay between peripheral and central sensitization, endogenous modulatory pathways, and cortical processing. For diagnosis of TMD pain a descriptive history, clinical assessment, and imaging is needed. However, due to the complex nature of pain an additional step is needed to render a definitive TMD diagnosis. In this review we explicate the role of different biomarkers involved in painful TMD. In painful TMD conditions, the role of biomarkers is still elusive. We believe that the identification of biomarkers associated with painful TMD may stimulate researchers and clinician to understand the mechanism underlying the pathogenesis of TMD and help them in developing newer methods for the diagnosis and management of TMD. Therefore, to understand the potential relationship of biomarkers, and painful TMD we categorize the biomarkers as molecular biomarkers, neuroimaging biomarkers and sensory biomarkers. In addition, we will briefly discuss pain genetics and the role of potential microRNA (miRNA) involved in TMD pain.

## Introduction

The orofacial region consists of heterogeneous hard and soft tissues that make diagnosing and treating pain conditions a challenging task. Pain of the orofacial region is debilitating and can arise from various structures innervated by the cranial nerves in particular trigeminal nerve. Majority of the orofacial structures transmit impulses through trigeminal pathways to the brain where pain is perceived as a subjective sensation by the dynamic interaction of cognitive, affective and sensory elements. Orofacial pain is a multidimensional experience that can affect the patient physically, emotionally, and impairs quality of life. The conditions associated with orofacial pain are broadly divided into different categories such as odontogenic, musculoskeletal, neurogenic, psychologic, headaches, and others. Clinically, due to diverse nature of pain complaints, it is difficult to distinguish many of the orofacial pain disorders that eventually leads to misdiagnosis, delay in diagnosis or ineffective management. It becomes more challenging for the clinician when the pain is persistent and devoid of any physical and or structural cause.

Recently, population-based cross-sectional surveys have shown that the prevalence rate of self-reported orofacial pain ranges from 10 to 26% and cost over 32 billion each year in the United States.^1–3^ Women are more frequently affected by a ratio of 2:1, and other described risk factors are low socioeconomic status, smoking, psychological factors and presence of other chronic pain conditions.^4,5^

In clinical settings, pain conditions are categorized as acute or chronic. In most instances, definition of chronicity varies among researcher and health care practitioner. According to the International Association for the Study of Pain “chronic” indicate persistence of pain beyond the normal time of healing, while for clinical practicability a duration exceeding 3 months and for research purposes, a duration of 6 months is used to denote the pain as being chronic.^6^

Acute pain is an adaptive, self-limited involving activation of nociceptors and chemical mediators with clearly defined cause. It may be very intense, often accompanied by anxiety and restlessness but the cause-and-effect relationship is usually apparent and treatment measures are usually effective. While chronic pain is persistent and resistant to treatment measures because of neuroplastic changes in the structures of central and peripheral nervous system. Presently, prevalence of chronic pain is increasing due to changes in work demands and increased symptom awareness and turn out to be major source of suffering as it interferes with daily functioning accompanied by distress, hopelessness, fatigue, depression and dependence. One of the chronic pain conditions which possess a global health problem is temporomandibular disorders (TMD).

**Table 1:** Summary of Potential TMD pain Biomarkers

Biomarkers type	Biofluids/Tissue source	Clinical significance	References
*Molecular biomarkers:* *Inflammatory cytokines*			
1) Interleukins: IL-1β, IL-6, IL-7, IL-8, IL-13, and MCP-1	Serum, Saliva and Synovial fluid	IL: Development and progression of TMD pain IL-1β, IL-6, IL-8 and MCP-1 involved in TMD pain secondary to disc disorders and degenerative joint diseases. IL-6 in transition from acute to chronic as well as IL-6 IL-7, IL-8, and IL-13 play role in TMD myalgia	Yaman et al.^27^, Sorenson et al.^29^, de Alcântara Camejo et al.^35^, Ogura et al.^33^, Shinodaet al.^36^, Alstergren et al.^32^
2) TNF	Synovial fluid	Primary role in TMD pain/Inflammatory connective tissue diseases	Guven et al.^43^, Fredriksson et al.^68^, Nordahl et al.^44^
3) Proteinases: MMP-1, MMP-2, MMP-3, MMP-7, MMP-8, MMP-9, MMP-13	Synovial fluid and trigeminal ganglion	Involved in TMD pain secondary to disc disorders and degenerative joint diseases.	Loreto et al.^50^, Tabeian et al.^48^, Gho et al.^49^, Huang et al.^54^, Nascimento et al.^55^, Puliti et al.^46^, Yoshida et al.^47^, Srinivas et al.^51^
4) Bradykinin PGE2, LTB4, F-2-Isoprostane,	Synovial Fluid and serum	Involved in TMD pain secondary to disc disorders degenerative joint diseases and muscle soreness.	Nishimura et al.^56^, Alstergren et al.^57^
*Neurotransmitters*:			
1) Glutamate	Synovial Fluid	Involved in pain processing, peripheral nociception, and central sensitization of painful TMD.	Miller et al.^59^, Alstergren et al.^61^
2) Serotonin	Synovial Fluid, Plasma and Saliva	Involved in Peripheral and cental pain mechanism of painful TMD. Sensitize neuropeptides and other neurotransmitters such as glutamate, as well as involved in the modulation and control of TMD pain.	Kopp et al.^67^, Fredriksson et al.^68^, Ernberg et al.^66^
3) Dopamine	Synovial fluid	In myofascial pain and degenerative diseases Pain perception as well as in motor control, cognition, and reward system	Dawson et al.^69^
Neuropeptides Substance P and CGRP	Synovial fluid and Saliva	In myofascial pain Reflect joint pain progression and pathogenesis.	Sato et al.^71^
Growth factors NGF and VEGF	Plasma and Synovial Fluid	Persistent pain and pain progression in TMD disc and degenerative joint diseases.	Chen et al.^74^, Ke et al.^75^
Epigenetic biomarkers (miRNA)	Tissue: Synovial fibroblasts and articular cartilage	Altered expression of miRNA221–3p, miRNA 140-5p, miR‐101a‐3p, miR21-5p were observed in DJD and Pain	Zhang et al.^81^, Li et al.^79^, Xu et al.^78^
Neuroimaging biomarkers	Structural MRI	Decreased trigeminal nerve fiber density, axonal diameter, myelination and GMV correlated well with TMD pain in patients with TMJ synovitis and myofascial pain.	Zhang et al.^91^, Wilcox et al.^87^, He et al.^98^, Moayedi et al.^88^, Gustin et al.^89^, Weissman-Fogel et al.^94^, Ichesco et al.^92^, Younger et al.^90^, Buckner et al.^93^
	Functional MRI	Alterations in FC, GMV CBF, MD was observed in different regions of high order cognition, emotion-related regions such as ACC, PCC, MCCC, mPFC, DLPFC, Amygdala, and PAG-raphe system.	
	Functional and Structural MRI	In motor system: structural and functional alterations, as well as changes in cortical processing such as increased cortical thickness or elevated activity, was observed in M1 and SMA areas	
	MRS	Neurochemical changes: alterations in NAA, Cho, and tCr levels were observed in patients with TMD pain	Harfeldt et al.^101^, Feraco et al.^105^, Fayed et al.^104^, Harris et al.^102,103^
QST	Sensory functioning	QST studies observed abnormalities in somatosensory profile as well as enhanced pain sensitivity in patients with TMJ arthralgia and myofascial pain	Wang et al.^116^, Zhou et al.^108^, Yang et al.^110,112^, Kothari et al.^111^
Pain genetics	SNPs in COMT gene	Glucocorticoid receptor gene-HPA axis, serotonin receptor gene-nociceptive afferent pathways, alpha subunit of the voltage-gated sodium channel Nav1.1-action potential in sensory nerves, prostaglandin-endoperoxide synthase 1 gene-nociceptive and inflammatory response, amyloid beta (A4) precursor protein- synapse formation and neuronal plasticity, PDZ domain protein gene-affects G protein-coupled receptors involved in nociception and analgesia. Serotonin receptor HTR2A, ERA as well as genetic and epigenetic factors such as SLC64A4, TRPV2, MYT1L, and NRXN3 along with environmental factors play a vital role in the development of chronic TMD pain.	D’Agnelli et al.^119^, Slade et al.^34,40^, Chen et al.^121^
Biochemical markers	Serum	Vitamin D and 8-hydroxydeoxyguanosine and malondialdehyde	Demir et al.^122^, Rodríguez de Sotillo et al.^123^

TMD are defined as a group of clinical conditions affecting the muscles of mastication, the temporomandibular joint (TMJ), and the related structures.^7^ TMD affects about 5%–12% of the general population,^7^ while some studies^8,9^ reported higher incidence of TMD as 25%–40% with women having greater risk of developing TMD conditions than men.^7^ It is the second most common musculoskeletal pain condition after chronic back pain.

Of all the painful orofacial conditions, most common conditions for which patient seek treatment are tooth-related pain and painful TMD. According to the recent classification on Orofacial Pain, temporomandibular joint pain (TMJ) indicates pain localized to the TMJ, occurring at rest or during jaw movement or palpation. TMJ pain is classified as primary pain which means pain that is not attributable to another disorder and secondary pain means pain caused by another identified disorder such as inflammation or due to infection, trauma, or autoimmune disorder, sensitization of the tissues, structural changes, muscle spasm, or injury. Secondary TMJ pain is diagnosed usually when the pain has developed in temporal relation to onset or substantial worsening of the presumed causative disorder or has led to its discovery or the pain has significantly worsened in parallel with progression of the presumed causative disorder which can be either disc disorders or degenerative joint diseases (DJD).^10^ In this review, we have used the term “painful TMD” which includes both primary and secondary pain disorders.

Painful TMD potentially occurs due to ongoing nociceptive input and interactions between peripheral and central sensitization, endogenous modulatory pathways, cortical processing and psychologic factors. Patient with TMD frequently report pain as the primary complaint that can occur from either intra-articular structures or from an extra-articular location as referred pain from adjacent structures. Other symptoms of TMD included joint noises, impaired jaw movements tinnitus, dizziness, cervical pain and headaches. TMD has multifactorial etiologies and consists of various contributing factors including trauma, either physical or emotional, biological process such as aging, postural condition such as abnormal head and cervical position, systemic predisposition, sleep disorders and psychosocial alterations.^11^ Genetic and sensory processing also contributes to the etiology of TMD. Recently, the Orofacial Pain Prospective and Risk Assessment (OPPERA) study reported that TMD is best viewed using a biopsychosocial model to emphasize the importance of psychosocial and behavioral factors to the onset of painful TMD.^12^

**Fig. 1:**
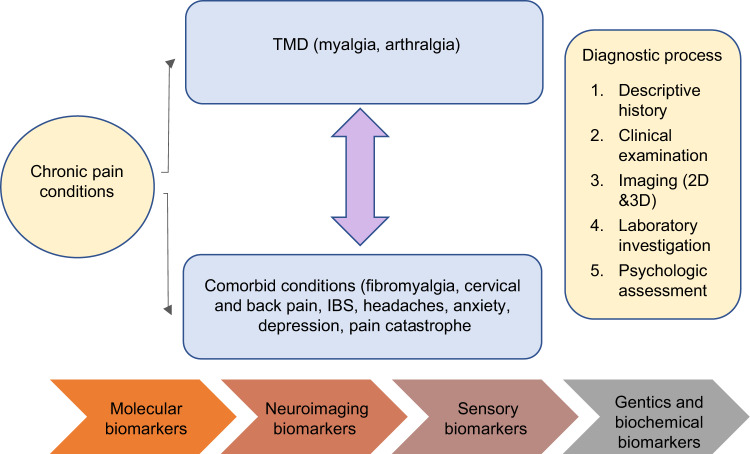
Diagnostic evaluation of painful temporomandibular disorders

Initially, it was observed that painful TMD are commonly caused by peripheral factors such as acute inflammation or trauma to the muscles, joint or adjacent structures. However, later it was noticed that not all patients have clearly identifiable peripheral etiological factors and lack of concordance was noticed between TMD and pain.^13^ Further, epidemiological studies show that comorbidities are common among individuals with TMD pain. It includes headaches, widespread pain and fibromyalgia, neck and back pain, the most common psychosocial disturbances such as stress, anxiety, depression and pain catastrophe and other visceral comorbid conditions such as irritable bowel syndrome and pelvic pain.^14,15^ These comorbidities not only co-occur with TMD but also contribute to onset and persistence of pain. Therefore, for diagnosis of painful TMD conditions, a special attention should be given to the history and clinical assessment. A descriptive history is required to render TMD diagnoses and identify the contributing factors. In some cases, clinical examination and history are not reliable and provide limited diagnostic information.^16^ So, diagnostic imaging is needed, in some cases to render a TMD diagnosis and to rule out other pathology. Moreover, several issues have emerged at the diagnostic level of TMD due to heterogeneity of the disorder. Also, association of multiple pain conditions such as fibromyalgia and back pain with TMD in a single patient or presentation of single pain complaint in multiple ways like referral pain in TMD and other orofacial pain conditions make this disorder more complex.^15^ Also, the underlying mechanism differ in each pain conditions and it is hard to identify risk factors in painful TMD as symptom of pain varies with time that depends on the chronicity of the condition. Additionally, differences in the TMD pain between the individuals at diverse points of time contribute to more challenges in diagnosis as well as in management of the TMD. Due to this inherent complexity of painful TMD and poorly understood etiologies making diagnoses and subsequent treatments exceedingly difficult. With a significant proportion of the population suffering from painful TMD, the development of new and accurate diagnostic procedures is essential to improve the current standard of care. (Fig. 1 shows different diagnostic methods used for diagnosis of painful TMD)

Biomarker research is a potential diagnostic method that has grown significantly in recent years. According to FDA biomarkers are “a defined characteristic that is objectively measured as an indicator of normal biological process, pathologic process or biological responses to a therapeutic intervention.^17^ The biomarker is a chemically stable molecule that can be easily quantifiable in the patients and could be used to predict the onset, persistence, severity, and prognosis of the patient’s pain state.^18^ There are various methods have been used for categorizing the pain biomarkers based on pain mechanism and disease characteristic. Researchers have also classified the pain biomarkers based on the method of acquisition such as direct physiological measures, bio samples, genetic testing, and imaging.^19–21^

For the study of painful TMD biomarkers, various steps have been identified such as discovery of specific biomarkers involved in pain, its validation, and clinical use.^22^ The clinical validation step assesses the sensitivity and specificity of the biomarker to identify, measure or predict the clinical outcome of the disorder which in this case is painful TMD. In addition, an ideal biomarker should be easily available, less expensive, and express the relationship between risk factors and diseases. It should be notable early in disease progression, consistently reliable, and validated in multiple populations using different clinical studies.

In painful TMD it is important to recognize the type of biomarker involved in specific pain condition which in this case can be diverse. For example, In TMD, myalgia and arthralgia are common painful conditions that frequently coexisted with other TMJ disorders such as intra-articular disc derangements, degenerative joint diseases, and oromandibular movements disorders. Similarly, in epidemiological studies direct causal relationship between two variables were assessed using measures of regression coefficients and odds ratio.^23^ However, due to confounding it is hard to find the causal relationship between two variables. For example, if patient anxiety increases the levels of certain biomarkers as well as increases the value of reported pain then association between biomarkers and pain is not causal. As in this case, anxiety provides the real causal mechanism for pain increase and biomarkers could be associated with anxiety. In such cases ideally biomarkers should be associated with the pain and not with the underlying disorders, but it is hard to find due to high variability among clinical conditions and pain presentation of individual patient. Therefore, combined biomarkers are more highly predictive of outcomes than individual markers. It may be appropriate to study multiple biomarkers, before validating and implementing the biomarkers for clinical use.

**Fig. 2:**
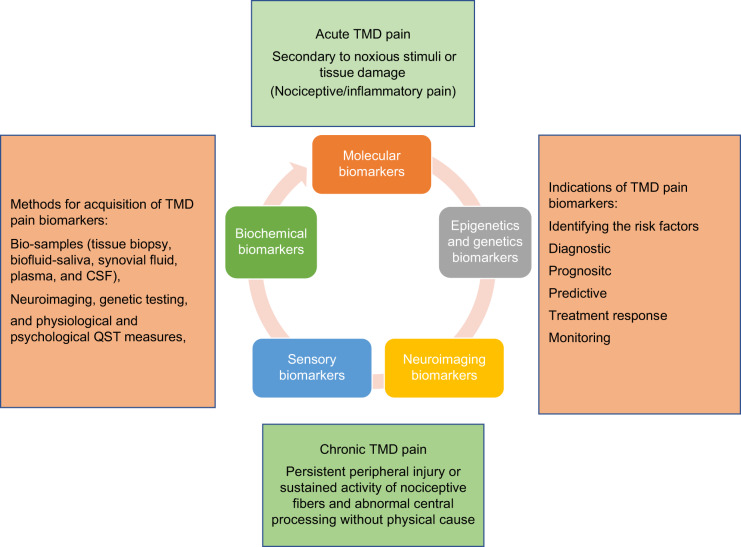
Relationship between TMD pain and biomarkers

In oncology and internal medicine biomarkers have been developed to a considerable point of structural, biochemical, and pathological mechanism while in painful TMD has conditions still there are very few biomarkers.^19^ Despite of the significant effort from researchers in identifying different biomarkers involved in painful TMD conditions, still there are currently no biomarkers specific biomarkers for TMDs. Most studies have focused on the relationship between TMD biomarkers and cross-sectional outcomes such as subtype degenerative joint diseases and inflammatory markers while some predicting longitudinal outcomes such as genetic polymorphisms and TMD.^12,24,25^ However, the identification of biomarkers in painful TMD has been afflicted by lack of validation in replication cohorts, cross sectional studies and inconsistent biomarker platforms, leading to discrepant reports. Current researchers are focusing on combination of biomarkers with acceptable specificity and sensitivity in order to obtain an individual patient’s pain profile. Therefore, most biomarker studies have been limited in assessing the relationship between biomarkers and painful TMD.^26^ (Fig. 2 shows the relationship between TMD pain and Biomarkers). We aim to explore different biomarkers involved in painful TMD. In this review, we are familiarizing the reader with biomarkers as molecular, neuroimaging, and sensory biomarker (Quantitative Sensory Testing-QST). Additionally, we are also briefly discussing about the role of miRNA, pain genetics, and biochemical markers in painful TMD.

## Role of Molecular Biomarkers in TMD pain

In general, biomarkers molecular biomarkers are analyzed using bio samples such as tissue biopsy and biofluid plasma, saliva, synovial fluid, and cerebrospinal fluid. Previously, blood serum is the gold standard for biomarker assessment and validation. In recent years saliva and synovial fluids have been the interest of researchers and clinicians due to the noninvasiveness of saliva collection and the apparent tissue specificity of synovial fluid. Synovium, the semipermeable boundary lining the synovial joint, naturally restricts the passage of oversized molecules, thereby preventing select joint-specific biochemical markers from entering the bloodstream. Hence, synovial fluid analyses can uniquely pinpoint joint conditions providing real-time information concerning joint which is not feasible through the evaluation of serum and saliva.^23^ Therefore, we have included majority of the studies which have used synovial fluid for analysis of biomarkers in TMD patients. However, a growing number of investigations also concluded that saliva and serum-based biomarkers are also accurate in differentiating pain patients from non-painful healthy participants.

The search for molecular markers in painful TMD has mostly focused on underlying inflammatory response such as markers in rheumatoid or osteoarthritis, disc derangements, and muscle disorders as myalgia myofascial pain and tendonitis. Yet, etiology and pathophysiology behind degenerative diseases, disc derangement, and muscle disorders are not clear. Emerging data suggest that inflammation or release of inflammatory cytokines possibly due to mechanical overload, local hypoxia, micro trauma, and reduced adaptive capacity of cartilage are suggested as mechanism in the pathogenesis of painful TMD.^23,25^

Therefore, investigators reported that inflammatory cytokines and cartilage degrading biomarkers such has prostaglandin (PG) and matrix metalloproteinases (MMP) are common biomarkers involved in individuals with painful TMD.^25,26^ Similarly, in chronic TMD states, in addition to inflammatory cytokines other molecular markers in form of neurotransmitters and neuropeptides have been observed. It is observed that peripheral tissue injury or nerve dysfunction can alter the status of neuropeptides and neurotransmitters that often lead to prolonged sensitization of peripheral pathways.^27^ Additionally, role of these molecular neurotransmitter and peptides have been determined in mechanism of central sensitization which means amplification of pain by abnormally amplifying ascending peripheral input or dysregulation of inhibitory modulatory system.^28^ Based on the involvement of these cytokines and neuronal molecules in painful TMD, we have categorized the molecular biomarkers as inflammatory cytokines including proteinases, neurotransmitters, neuropeptides, and growth factors.

### Inflammatory cytokines

Cytokines are small peptides that are released during inflammation and have both pro- and anti-inflammatory effects (proinflammatory biomarkers Interleukin 1 beta (IL- 1β), IL-6, TNF, and IL-8 and anti-inflammatory biomarkers IL-4 and IL-10). Since these cytokines are studied in inflammation their potential role in pain secondary to disc displacements, DJD and muscle disorders have gained considerable interest.^28^ However, it should be noted that an ideal biomarker in painful TMD should have high sensitivity and specificity and can easily be quantified before obvious clinical symptoms are present in patients and during or after treatment.

#### Interleukins

To date, three subtypes of IL-1 have been identified such as inflammatory IL-1α and IL-1β, as well as IL-1 receptor antagonist, IL-1ra. IL-1 induces several inflammatory events and plays a vital role in hyperalgesia through activation of lymphocytes, stimulation of other cytokines, prostaglandin, and collagenase release from connective tissue cells. IL-1 family have a significant impact on TMJ homeostasis.^29^ There are studies that have investigated the potential role of IL in which pain occurs secondary to DJD, disc, and muscle disorders. In a review, the synovial fluid was analyzed in patients with painful DJD. Researchers observed that increased cytokines, IL-1β typically result in the development and progression of painful TMD.^30,31^ In contrast, some studies reported high synovial fluid IL-1ra and low IL-1β concentrations in patients with resolution of arthritis.^32^ In a study Ogura et al., noticed that IL-1β upregulate monocyte chemoattractant protein-1 (MCP-1) in serum and saliva which is chemokine and considered to be involved in recruitment of monocytes to the TMJ synovium in degenerative joint diseases patients.^33^ Also, MCP-1 is expressed in sensory neurons, and its expression in both neurons and peripheral tissues is upregulated following local inflammation.^33^

Likewise, Slade et al. observed high concentration of IL-1 and IL-8 in serum and saliva in patients with widespread pain and TMD.^34^ Similarly, studies^35,36^ have shown a relationship between inflammatory process and destruction of TMJ synovium and IL-6 has been pointed out as one of the most important proinflammatory cytokines contributing to the pathogenesis of disc disorder and degenerative changes. IL-6 is a pleiotropic cytokine produced by produced by variety of cells such as synovial cells, endothelial cells, macrophages, T-cells, smooth muscle cells, and fibroblast in the TMJ. It plays an important role in transition from acute to chronic pain inflammation and presents a dual effect with both anti and proinflammatory effect. In a study, level of IL-6 was observed higher in the synovial fluid of patients with TMJ pain with DJD compared to healthy controls which states that high IL-6 levels are associated with pain.^35,36^ It is noteworthy that IL-8 is another proinflammatory cytokine consisting of multifunctional actions in severe and critical inflammation.^37^ Sato et al., also found a high association between the levels of IL-6 and IL-8 in the synovial fluid of the patients with disc disorders and degenerative changes, indicating that a collaborative activation of theses cytokines is possible in TMD.^38^ In addition to synovial fluid analysis, intra-muscular cytokines and salivary biomarkers have also been studied in TMD. In a study, an elevated level of IL-6, IL-7, IL-8, and IL-13 have been observed in the masseter muscles of patients with TMD myalgia.^39^

In another study, Slade et al. observed the role of cytokines MCP-1, IL-1ra, and IL-8 in TMD with and without widespread pain. They observed odds ratios of 1.5 for MCP-1 and 1.4 for IL-1ra which signified that an increase in concentration of each cytokine was associated with an increase of ~50% in odds of TMD without widespread pain. Also, high levels of MCP-1 and IL-8 increased odds of TMD with widespread pain, with no independent contribution of IL-1ra was observed. They concluded that TMD without widespread pain cases had elevated levels of the proinflammatory cytokine MCP-1 and the anti-inflammatory cytokine IL-1ra. In contrast, TMD with widespread cases had elevated levels of the proinflammatory cytokine IL-8 with no compensatory increases in IL-1ra, thus indicating a more severe pro-anti-inflammatory imbalance among TMD with widespread cases.^40^

Overall, above cross-sectional and case-control studies showed that pain in TMJ is associated with inflammation secondary to arthritis or disc disorders and molecular markers such as IL-1β, IL-6, and IL-8 plays an important role in the onset and progression of the disease. These markers can be used to predict the outcome and progression of painful TMD. These studies also reported that IL-1β, IL-6, and IL-8 have high sensitivity and high specificity to painful TMJ with underlying inflammation. However, it is still unclear which specific markers needed to be assayed.

#### Tumor necrosis factor (TNF)

TNF is synthesized by many cells such as macrophages and monocytes, T cells, B cells, and fibroblasts. TNF can also upregulate its own synthesis and rapidly induces synthesis of other mediators such as IL-1, IL-6, and prostaglandins. Conversely, several cytokines such as endogenous immunosuppressive cytokines IL-4 and IL-10 and inhibitors of prostaglandin synthesis and glucocorticoids downregulate TNF expression.^23^ Interestingly, high concentrations of TNF also observed in dorsal root ganglion neurons and peripheral afferent fibers which explains its specific role in the peripheral mechanism of pain.^41,42^

In a study, synovial fluid TNF levels have been shown to be significantly higher in individuals with TMJ pain upon mandibular movement than in those without such pain.^43^ Nordahl et al., reported that the patients with chronic inflammatory connective tissue disease and associated TMJ pain have elevated TNF levels in the synovial fluid compared to those without pain.^44^ Also, in a clinical study a high pretreatment level of TNF in the TMJ synovial fluid was found to be associated with TMJ pain. However, after treatment with glucocorticoid intraarticular injection, a reduction of synovial fluid TNF and pain relief was observed.^45^ These studies explain that TNF has high sensitivity in inflammatory diseases and pain that can be used as a marker to understand the peripheral mechanism of pain and to assess the progression of painful TMJ disorders.

#### Proteinases

Based on the discussion of IL and TNF it could be considered that underlying inflammation in patients with TMD is one of the potential sources of pain. Another family of cytokines called MMPs are discussed here. MMPs are group of enzymes that are released during inflammation and associated with tissue destruction. MMPs are proteolytic enzymes that degrade extracellular matrix components and has been noticed to be involved in TMJ inflammation and pain modulation.^24^

After exploring the literature, it has been noticed that the expression of MMP-1, MMP-2, and MMP-13 increases in joint inflammation which contributes to progressing of degenerative changes and pain.^46–48^ Also, in few other studies, in patients with disc disorders and degenerative joint changes an increased level of collagenases (MMP-1, MMP-8, MMP-9, MMP-13), Stromelysin (MMP-3), and gelatinases (MMP-2 and MMP-7) in TMJ synovial fluid was observed.^49–53^

In pre-clinical models Huang X et al., observe the effect of psychological stress on TMJ of rats. They noticed an increased expression of MMP-3 correlated well with degree of TMJ lesion.^54^ Also, Nascimento et al., analyzed the expression of MMP-2 and MMP-9 in the rat trigeminal ganglion during the development of TMJ inflammation.^55^ In this study researchers evaluated whether mechanical allodynia orofacial hyperalgesia, induced by the injection of complete Freund’s adjuvant into the TMJ capsule, were altered by an MMP inhibitor (doxycycline, DOX). They observed MMP ex-pression in the trigeminal ganglion was shown to vary during the phases of the inflammatory process. MMP-9 regulated the early phase and MMP-2 participated in the late phase of inflammatory process. Additionally, the increases of mechanical allodynia and orofacial hyperalgesia were attenuated by the with DOX a nonspecific MMP inhibitor.^52^ In painful TMD research, MMPs are gaining a considerable interest and it can be used as a therapeutic target for management of pain. Although multiple molecular biomarkers have found to be above than normal concentrations in painful TMD, it is still unclear how specific mediators should be chosen. Also, the concentrations of cytokines vary considerably in individuals with painful TMD. Therefore, multiple biomarkers should be assayed to understand the complexity of painful TMD.

#### Other molecular biomarkers

Bradykinin is one of the inflammatory mediators and plays an important role in nociception and sensitization. It is a potent vasodilator and bronchoconstrictor that increases vascular permeability and facilitates pain transmission. In a study on TMJ disc disorders and DJD high bradykinin levels were observed correlated positively with the degree of inflammation^56^ Similarly, prostaglandin PGE2 produced by COX-enzyme during inflammation is also an important biomarker observed in pain studies and found to associated with in delayed onset muscle soreness.^23^ In addition, Alstergren et al., also detected increased levels of PGE2 in synovial fluid of patients with chronic inflammatory joint disease, and the levels were found to be related to TMJ pain on mandibular movement.^57^ Recently Basi et al., studied the biomarkers in TMD patients and controls.^58^ They collected venous blood, biopsied masseter muscles, and TMJ synovial fluid on the subjects’ side of maximum pain intensity with disc displacement and concurrent joint pain and myofascial pain. They assayed bradykinin, nerve growth factor, PGE2, LTB4, F2-isoprostane, and substance P and noticed that individual pain mediators analyzed were not correlated between plasma, muscle, synovial fluid, and painful TMD except for F2-Isoprostane.^58^

### Neurotransmitters

In painful TMDs, neurotransmitters play a key role in peripheral or central sensitization as well as central processing. Along with pain processing neurotransmitter have strong impact on mood and behavior. All the major neurotransmitters are inflammatory mediators included PGE2, PGI2, LTB4, NGF, Bradykinin, adenosine, substance P, 5-HT, histamine, glutamate, noradrenalin, NO and non-inflammatory mediators as CGRP, GABA, opioid peptides, glycine, and cannabinoids that are typically involved in inhibitory and facilitatory pain mechanism. Currently, monoamine neurotransmitters such as glutamate, serotonin (5-HT), and dopamine have been explored as potential biomarkers in painful TMD conditions.

#### Glutamate

Glutamate is the most abundant excitatory neurotransmitter present in afferent sensory nerves for conveying sensory information to the central nervous system (CNS). It is present in the trigeminal ganglion as well as in the central and peripheral inflammatory sites. Typically, the sensory neurons contain full set of glutamate receptors including NMDA (N-methyl-D-aspartate), AMPA (α-amino-3-hydroxyl-5-methyl-4-isoxazole-propionate) which are the potent modulators of central sensitization.^59^

In a study, researchers noticed an elevated level of glutamate in the joints with inflammatory conditions such as degenerative diseases secondary to rheumatoid arthritis. They reported that glutamate usually act as a mediator and is not primarily associated with inflammation whilst its receptors have been found to possess modulatory roles in peripheral nociception and sensitization.^59–61^ This finding suggest that glutamate can arise from nerve fibers in the inflamed synovial membrane or from plasma extravasation into the synovial tissues and can be used to understand the underlying mechanism of peripheral and central sensitization specifically in patients with painful TMD. This also means that glutamate has high sensitivity and specificity towards painful TMD. It is also noticed that glutamate injection in the healthy TMJ evokes immediate pain that is partly mediated by peripheral NMDA receptors in the synovial tissues. In order to overcome TMJ pain, researchers tried to peripherally block the NMDA receptor by ketamine or NMDA antagonist and noticed partial reduction in pain.^61^ This observation explains the role of glutamate in pain processing. However, additional studies are needed to understand the role of glutamate in pain behavior in painful TMDs.

#### Serotonin

Serotonin or 5-hydroxytryptamine (5-HT) modulates physiological processes in both the central and peripheral nervous systems. It is synthesized both peripherally and in the CNS from the essential amino acid tryptophan, which is derived from the diet. In the CNS, 5-HT is found in serotonergic neurons and considered to have an important role in control of pain through descending inhibition. However, outside of CNS 5-HT act as an inflammatory mediator and sensitizes afferent nerve fibers to induce hyperalgesia.^62^

It is observed that in response to tissue trauma and inflammation, 5-HT3 a subtype of 5-HT is released and sensitizes peripheral neurons which seems to be important for mediating pain from the periphery.^63,64^ Previous research noticed an increase number of 5-HT3 receptors on sensory nerves of the masseter muscle in patients with myalgia compared to controls which showed that serotonin is involved in in myalgia.^65,66^ 5-HT also sensitizes peripheral mechanoreceptive afferent fibers to other chemicals such as, glutamate, Substance P (SP), Calcitonin gene related peptide (CGRP) by enhancing the efficiency of tetrodotoxin-resistant sodium channels and lowering the threshold of TRPV1 receptors, which results in primary hyperalgesia.^67^ This explains that in patients with hyperalgesia serotonin can be used a biomarker. In TMJ of healthy individuals’ serotonin is usually undetectable in synovial fluid which is important from a diagnostic point of view. In a study, investigators observed an elevated serotonin levels in the TMJ synovial fluid that has been strongly associated with TMJ pain provoked by functional mandibular movements.^65^ Several other studies have demonstrated that patients with chronic myalgia and myofascial pain have significantly higher level of 5-HT in saliva and serum compared to control and high levels are correlated with allodynia.^65–67^ In another study, in patients with fibromyalgia, elevated synovial 5-HT has been observed to be associated with increased pain and high levels of anxiety.^66^ In another clinical study, researchers investigated the influence of 5-HT on the effects of intra articular injections of glucocorticoid on pain of the TMJ in patients with inflammatory disorders of TMJ. Researchers concluded that both local and systemic levels of 5-HT partly determine the effect of intra-articular glucocorticoid injection on TMJ pain in patients with chronic arthritis or degenerative diseases.^68^ From the above-mentioned studies, it could be inferred that serotonin plays a vital role in in peripheral and central pain mechanism and highly predictive of painful TMD compared to other biomarkers.

#### Dopamine

In addition to 5-HT, Dopamine is also involved in pain perception as well as in motor control, cognition, and reward system. Primarily dopaminergic neurons synthesize dopamine in the CNS while adrenal medulla and neuroendocrine cells synthesize dopamine in the peripheral nervous system. A more recent study found no difference in plasma levels of 5-HT between patients with myofascial TMD and healthy controls, but that plasma dopamine levels were significantly increased in TMD patients.^69^ These findings suggest that peripheral dopamine might be involved in modulating peripheral pain and a complex relationship existed between the peripheral and central actions of these neurotransmitters in TMD pain.

### Neuropeptides

Neuropeptides are small peptides that are released from neuronal cells and are commonly used by neurons to communicate with each other. Many neuropeptides are co-released with other neurotransmitters, such as glutamate, acetylcholine, and norepinephrine. Some neuropeptides are released peripherally and have a key role in neurogenic inflammation, such as SP, CGRP), etc. whereas others are released in central regions, such as galanin, cholecystokinin, and neurotensin. It is observed that CGRP and substance P are widely produced both in central and peripheral nervous system and can amplify variety of inflammatory and nociceptive processes.^70^

In a study of painful TMD patients, CGRP levels are positively correlated with pain and SP have been found to be increased in synovial samples with no positive correlation.^71^ These observations suggest that SP levels may reflect joint injury or pathology, whereas CGRP levels may be more reflective of joint pain. However, it is not clear whether these associations reflect increased expression in response to mechanical joint injury, or whether these neuropeptides are secreted in response to local tissue hypoxia that may occur in TMD.^72,73^ Therefore, more studies are needed to elucidate the complex relationship between SP, CGRP levels and pain. In addition to synovial fluid as medium for analysis, saliva has also been employed as an indicator of stress and chronic pain. For example, reports state that substance P, a neuropeptide associated with inflammation and pain, the stress hormone cortisol, and markers of oxidative stress can be repeatedly detected within salivary secretions. Suggesting a preference for saliva over serum in the detection of select markers, researchers go on to describe that substance P is actually more readily available in oral fluids than patient-matched blood samples.

### Growth factors

The nerve growth factor (NGF) belongs to the family of neurotrophins, a well-known mediator for persistent pain. In general, NGF is expressed after the injury or inflammation to the TMJ, and initiates signal cascades at the peripheral sensory neurons. This cascade involves activation of the PI3K-AKT pathway and ERK-phosphorylation, as well as activation of the protein kinases PKCγ and PKA, leading to sensitization of ion channels, such as TRPV1 that subsequently enhanced nociceptive activity and inflammatory pain.^73,74^ Additionally, NGF also produces hypersensitization and elevated NGF levels were found in the plasma of patients from persistent inflammatory pain states, such as bladder pain syndrome chronic prostatitis and chronic migraine. Moreover, increased NGF was found in synovial fluid of patients with DJD secondary to RA, and cancer pain.^73^

In addition, vascular endothelial growth factor (VEGF) produced by endothelial cells, chondrocytes and osteoclast have also found increased in condylar cartilage of mechanically induced DJD and in synovial fluid and tissue in patients with disc disorders associated with tissue degeneration.^75^ Other factors such as brain-derived neurotrophic factor, fibroblast growth factor, insulin like growth factor-binding protein and transforming growth factor were detected in TMJs with disc disorders and degenerative joint diseases.^24^

Although many studies have used synovial fluid for analysis, along with saliva and serum these biofluids still provide an invaluable source of biochemical information. Also, acquiring synovial samples is accompanied by a degree of invasiveness based on which it can be inferred that at this time there is no one ideal biofluid capable of imparting all aspects of the TMD. While serum, saliva, and synovial fluids all contain biomarkers indicative of unique disease states, both local and systemic, none are considered comprehensive, hence necessitating the ongoing research for personalized diagnostics and therapeutics.

## Role of miRNAs in TMD pain

To understand the pain chronification one area which has drawn the attention of many researchers is epigenetics in chronic pain. It is noticed that microRNAs (miRNAs) a class of small non-coding inhibitory RNAs play an important role in regulating pain processing within a wide range of experimental models and clinical pain disorders. miRNA is a novel class of non-coding single-stranded RNA of 19–24 nucleotides with the ability to modulate a large proportion of the genome post-transcriptionally.^76^ They bind to the 3′ untranslated region (UTR), or occasionally 5′ UTRs, of the multiple mRNA targets to which they exhibit imperfect, or sometimes, perfect complementarity. This enables one specific miRNA to inhibit the translation of multiple genes.^77^ Also, it is established that miRNAs are essential regulators of many proteins involved in proper neuronal function and takes part in the morphological and functional changes sustaining neuronal plasticity.^76,77^

So far there are a few published studies evaluating these biomarker profiles in TMD. In a study, a decreased expression of the miRNA221–3p was observed in synovial fibroblasts from patients with degenerative joint diseases. The researchers found that miRNA221–3p inhibits transcription of Ets-1, which is itself a transcription factor for MMPs enzymes responsible for tissue degradation and re-modeling in the joint cartilage.^78^ In another study, researchers evaluated the miRNA 140-5p that is expressed in TMJ DJD. They suggested that miR-140-5p may play a role in regulating mandibular condylar cartilage homeostasis and potentially serve as a novel prognostic factor of TMJ degenerative changes.^79^ In other few studies the biological function of the micro–ribonucleic acid 101a‐3p (miR‐101a‐3p) and miR21-5p (MiR21) in TMJ degenerative changes were explored.^80^ Researchers suggested that both miR‐101a‐3p and miR21-5p was involved in the cartilage matrix degradation as well as in progression of degenerative changes of TMJ.^81^

From the above results, it could be suggested that further studies on miRNA are needed which produce the same results to understand the exact involvement of miRNA and painful TMD.

**Fig. 3:**
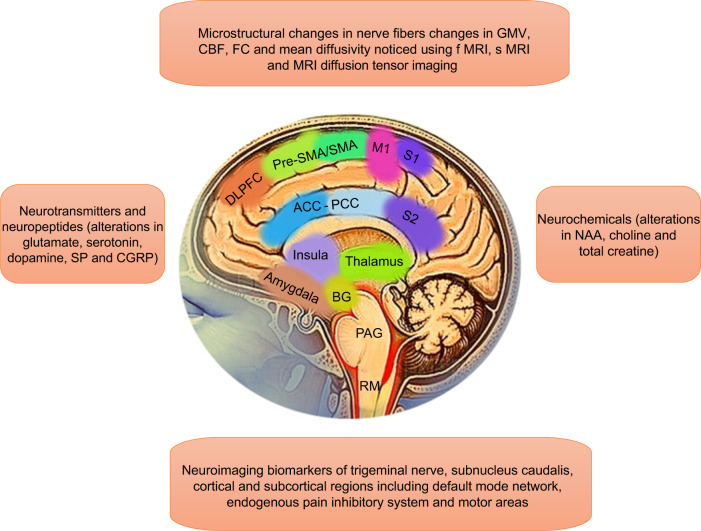
Neuroimaging markers in painful temporomandibular disorders

## Role of Neuroimaging markers in TMD Pain

Although it is observed that peripheral and central mechanism are involved in the pathogenesis of painful TMD, the neurobiological mechanism pertaining to pain remains to be clarified.^82–84^ Researchers observed that increased excitability in central nociceptive mediating neurons and centers associated with pain as well as decreased activity in the descending inhibitory system seems to be involved in process of painful TMD.^85^ From this observation it could be anticipated that there are certain areas in the brain that specifically show alterations in painful TMD and can be used as a Biomarkers. Therefore, to explore those biomarkers investigators have used an enticing method called neuroimaging.

Neuroimaging investigates the brain function, structures, and changes in neurochemicals which can be used as a potential biomarker in painful TMD. Functional and structural magnetic resonance imaging (fMRI) and (sMRI) and magnetic resonance spectroscopy (MRS) methods have been widely used separately or combined to explore brain alterations in patients with chronic pain.^86^ Also, one very interesting technique that has been recently applied to assess white matter as well as in grey matter volume in the brain of TMD patients is MRI diffusion tensor imaging/fractional anisotropy combines with sMRI. These techniques also have demonstrated changes in massive reorganization and functional connectivity of the spinal, subcortical and cortical structures involved in pain perception and modulation in patients with neuroplastic changes or chronic pain conditions like TMD.^87^

Neuroplasticity is thought to occur following continuous barrage of nociceptive afferent signals to the brain. Hence, TMD pain is presumed to induce anatomical and physiological changes in the brain over time. Inherent risk factors which give rise to onset of chronic pain, may also contribute to brain abnormalities present in patients. Therefore, in this paper, we are familiarizing the reader with studies that have investigated the neuroimaging biomarkers of painful TMD patients in different regions of brain such as ascending pain system, lateral-medial pain systems, default mode network and antinociceptive descending pain modulations system and motor system Fig. 3 depict different Neuroimaging markers involved in painful temporomandibular disorders. In general, researchers are developing biomarkers to understand the TMD pain mechanism, diseases progression, and predict response to medication.

There are structural MRI studies that have compared TMD patients and controls and found evidence of decreased nerve fiber density, axonal diameter and myelination as well as microstructural changes in response to nociception in trigeminal nerve roots.^87^ Similarly, investigators also noticed an increased cerebral blood flow and decreased grey matter volume (GMV) in the ipsilateral spinal sub nucleus caudalis (SpVc) which processes nociceptive input of the TMD patients. They reported that reduction in grey matter volume reflect neuronal loss, while the elevated blood flow in SpVc could be a compensatory response of increased neural activity to structural reductions or hyperexcitability due to nociceptive pathways.^88^

In another study, GMV has been assessed in thalamus. Investigators observed a positive correlation between thalamus GMV and pain duration.^88^ This potentially due to persistent trigeminal nociceptive input, which further contributed to the hyperalgesia of TMD by enhanced facilitating trigeminal sensory information in thalamus. These findings suggest that micro structural changes of the nerve in form of fiber density and GMV can be used as biomarker to understand the complexity of painful TMD mechanism. However, it seems like these neuroimaging findings have low sensitivity and reproducibility so further studies are needed to validate these findings.^89^

Also, there are studies that observed different cortical regions using fMRI. Investigators have studied lateral pain system which is involved in encoding the pain intensity as well as somatotopy. Also, medial pain system implicated in mediating the more affective-motivational aspects in the experience of pain have been evaluated. In a study Younger et al. investigated female patients with myofascial TMD and reported increased GMV in the right anterior insula and a negative correlation between self-reported pain intensity and GMV in the anterior cingulate cortex (ACC) and posterior cingulate cortex (PCC) were observed.^90^ In another study using fMRI Zhang et al. found decreased regional homogeneity in the right anterior insula in female patients with TMJ synovitis pain relative to healthy controls.^91^ Zhang et al., also reported decreased functional connectivity (FC) between median cingulate cortex (MCC) and anterior insula as well as decreased FC between MCC and dorsolateral prefrontal cortex (DLPFC) which was negatively correlated with pain intensity in TMD patients, which means patients with decreased connectivity reported higher pain.^91,92^

Furthermore, default mode network (DMN) is a group of functionally interconnected brain regions that get activated during mind wandering and not involved in any specific task and becomes correspondingly deactivated during goal-oriented tasks has been studied in previous studies.^93^ In a study by Weissman-Fogel et al., observed a functional disconnection within the DMN of prefrontal cortex (mPFC) and posterior cingulate cortex (PCC) in TMD patients with emotional interference.^94^ From these studies it is implicated that mPFC and PCC are two functionally connected regions processing introspective thoughts which is required for coping TMD pain. Also, in another study pain rumination scores which means repetitive negative thinking about pain were found to be positively correlated with the FC between mPFC and PCC, and periaqueductal gray PAG.^95^ This implies that TMD pain alters the normal function of these circuits which affects pain perception. Further, a key region of the endogenous pain inhibitory system PAG is well-positioned to modulate pain perception for interactions between ascending inputs from peripheral tissue and descending projections from brain regions such as ACC and mPFC and shows anatomical alterations in TMD pain conditions.^96^ Wilcox et al., demonstrated that in TMD participants, the PAG displayed a significant increase of mean diffusivity (MD) value and no GMV change, while the nucleus raphe magnus showed GMV decrease and no change in diffusivity.^87^ In another study using sMRI to assess the cortical thickness. Researchers reported that TMD patients had thicker cortex in the frontal pole and ventrolateral prefrontal cortex compared with healthy controls, and that cortical thickness of orbitofrontal cortex was negatively correlated to pain unpleasantness^88^ while cortical thickness in the left ventromedial prefrontal cortex was positively correlated with neuroticism scores in TMD patients.^97^

Investigators have also assessed the motor system in patients with TMD pain.^98,99^ Wessman-Fogel et al., found that TMD patients showed elevated activity in the primary motor cortex (M1) and supplementary motor areas (SMA) during the cognitive interference.^94^ Furthermore, in an sMRI study by Salomons *et al*., observed self-reported helpless-ness in TMD patients assessed by Pain Catastrophizing Scale was positively correlated with the cortical thickness of SMA which is a critical region implicated in cognitive aspects of motor behavior.^99,100^ However, further analysis identified neither significant group difference in the cortical thickness of SMA nor correlation with pain characteristics.^99^

Overall, the observation that certain brain areas are activated by transient painful stimuli in TMD patients, and that pain intensity is used for grading the magnitude has prompted researchers to understand brain activity that could serve as biomarkers to measure pain objectively. However, most of the brain responses observed when pain is present can also be observed when pain is absent. For example, similar brain responses can be elicited by non-painful auditory, tactile and visual stimuli, and such responses can even be recorded in patients. Therefore, there is still disagreement on the degree to which current measures of brain activity exactly relate to TMD pain. Furthermore, whether more recent analysis techniques can be used to identify distributed patterns of brain activity specific for pain can be only warranted using carefully designed control conditions. Also, the clinical utility of current pain biomarkers derived from structural and functional neuroimaging appears to be overstated, and evidence for their efficacy in clinical conditions is scarce. Initial results were promising need to be further tested for replicability and generalizability.

### Neurochemicals as a biomarker in TMD pain

Several studies have indicated that TMD pain conditions are associated with changes in brain Metabolism.^101^ In these studies, MRS imaging was used to assess neurochemical metabolites. It includes are N-acetyl aspartate (NAA), choline (Cho), and total creatine (tCr) as well as other metabolites such as glutamate (Glu), glutamine (Gln), and myo-insitol.^101^ According to the previous literature NAA is a biomarker of neuronal health and axonal numbers, while Cho is associated with in-creased cell numbers, membrane synthesis, or membrane breakdown. tCr is considered an important marker of cell energy and cell metabolism. Glutamine is a metabolite of glutamate and together they participate in complex metabolic activity cycles and intercellular communication involving neurons and astrocytes.^102,103^ MI is primarily present in glial cells and plays an important role in osmoregulation.^104,105^ It is observed that few of these neurochemicals can be used to assess the underlying mechanism of TMD Pain.

In a study, Gerstner et al., found a negative correlation between Gln levels in the left insula and pain in TMD patients while NAA and Cho levels in the left posterior insula were increased compared to healthy controls. In this study NAA levels were positively correlated with the duration of pain.^106^ Similarly, Harfeldt et al. reported elevated tCr levels within the posterior insula in TMD patients relative to healthy controls. Likewise, in the same study, increased Cho levels correlated with a reduced capacity for mouth opening and lower pressure pain threshold on the hand, and Glu levels were positively correlated with temporal summation of the nociceptive mechanical stimulus.^101^ These findings provide new evidence about the involvement of the neurochemicals as a biomarker in the neurobiology of underlying TMD. It is also a further step towards understanding and accepting that biomarkers can be used to assess the pain mechanism. However, need to be further tested for replicability and validity. It is observed that few of these neurochemicals can be used to assess the underlying mechanism of TMD Pain.

## Sensory marker-quantitative sensory testing in TMD pain

Although the investigators have been working on eliciting the potential biomarkers in TMD pain, still the somatosensory changes underlying this disorder are not fully understood. Previous studies indicate that somatosensory abnormalities can play a vital role in the underlying pain mechanism of chronic TMD.^107^ Therefore, comprehensive psychophysical examination provides unique, sensitive, and specific information about the neurological deficits of neuropathy and neuropathic pain. These examination helps the clinician to quantify the function of nerves involved in pain transmission. One of the tests that detect the sensory functioning is quantitative sensory testing (QST). Quantitative sensory testing (QST) is a psychophysical test method that investigates the functional state of the somatosensory system of a patient with regard to the severity of clinical signs by means of calibrated stimuli and subjective perception thresholds.^108^ It determines normal and aberrant sensory parameters, such as mechanical, cold, or heat detection and pain thresholds in patients with orofacial symptoms.

However, QST rarely used in clinical practice as it requires the active participation of the individual, and wide variations between the patients so, it lacks the validity. Yet, when carried out in a strictly standardized condition, this method is reliable to assess sensory nerve function. In TMD most frequent somatosensory abnormalities are gain of function (hyperalgesia) to pressure, pinprick, cold and heat stimuli, and an increased temporal summation.^109–111^ In addition, different somatosensory profile can also be identified in subgroups of myofascial pain TMD.^110,112^ In a study investigator reported alterations in the QST profiles of some TMD patients compared to healthy controls and chronic widespread fibromyalgia patients suggesting that QST may be able to provide specific pain signatures that could be used to assist in the diagnosis of painful TMD.^113^ In another QST study of TMD patients have found that enhanced pain sensitivity is associated with subsequent development of TMD, and that TMD is associated with various abnormalities in somatosensory profiles compared to controls.^114,115^ Recently, multiple phenotypes of facial somatosensory abnormalities were detected in Chinese TMD arthralgia patients using QST despite the disappearance of clinical signs and symptoms using QST.^115,116^ Although various QST measures have been correlated with subjective pain in the previous case-control study still there is a lack of evidence in the literature for the use of QST as a clinical predictor of TMD. Therefore, more research is required to explore the relationship between QST parameters in patients with TMD pain conditions.

## Other potential biomarkers of TMD pain

In addition to major advances in the molecular and neuroimaging research, pain biomarkers have also been assessed using person’s genetic code called as pain genetics. It is reported that epigenetic regulation can modulate gene expression, altering gene function without disturbing the underlying genetic code.^116,117^ Thus, each step in the sequence from genetic code to gene expression is a potential source for biomarkers.

In the literature COMT gene enzyme catecholamine-O-methyltransferase that inactivates or catabolizes the neurotransmitters dopamine, norepinephrine (NE), epinephrine, as well as caffeine and estrogens has been well studied in the humans and rodents.^118,119^ As these neurotransmitters play important roles in processing nociceptive inputs, this gene has been the focus of a wide range of studies of diverse acute, inflammatory, and chronic TMD pain. COMT is expressed in several types of neurons and glia cells throughout the CNS and PNS and affects many neural functions such as behavior, cognition, and motor control.^120^

Similarly, single nucleotide polymorphism (SNPs) in the COMT gene have been assessed to understand the development of TMD pain. individual SNPs provide important insights into etiology, while complex conditions such as TMD are likely influenced by combined effects of multiple SNPs operating through specific biological pathways. In OPPERA study, three hundred genes were investigated, implicating six SNPs as risk factors for chronic TMD. One SNP was in the glucocorticoid receptor gene, suggesting a contribution of the hypothalamic-pituitary-adrenal system to chronic TMD. Another SNP was in the serotonin receptor gene, supporting other studies indicating that the gene influences nociceptive and affective pathways. Next was the gene encoding the alpha subunit of the voltage-gated sodium channel Nav1.1, which influences action potentials in sensory nerves. It was associated with nonspecific orofacial symptoms such as jaw stiffness and fatigue. Also, psychological and somatic symptoms were associated with an SNP in the prostaglandin-endoperoxide synthase 1 gene, also known as COX-1, which regulates nociception and inflammatory response has been noticed as well as variation in the gene encoding APP, amyloid beta (A4) precursor protein, which affects synapse formation and neuronal plasticity were found to be associated with psychological stress and negative affectivity. Finally, heat pain temporal summation was associated with an SNP in the multiple PDZ domain protein gene, which influences G protein-coupled receptors involved in nociception and analgesia has been assessed.^34^

In a study,^40^ Single nucleotide polymorphisms (SNPs) coding cytokines and transcription factors were genotyped. They observed that TMD cases with and without widespread pain. Researchers observed that TMD cases with and without widespread pain had inhibited transcription activity of the anti-inflammatory cytokine transforming growth factor β1 (TGFβ1). They reported interactions between TGFβ1 and IL-8 SNPs an additional copy of the TGFβ1 rs2241719 minor T allele which was associated with twice the odds of TMD cases with widespread pain among individuals homozygous for the IL-8 rs4073 major A allele, and half the odds of TMD cases with widespread pain among individuals heterozygous for rs4073. These results demonstrate how pro- and anti-inflammatory cytokines contribute to the pathophysiology of TMD and wide-spread pain in genetically susceptible people.^40^

In a study, Chen et al. reported that multiple physiological and psychological regulatory domains may contribute to the pathophysiology of pain in temporomandibular disorder (TMD) and other bodily pain conditions.^119^ They evaluated the relationship between multisystem dysregulation and the presence of TMD pain, as well as the presence of different numbers of comorbid pain conditions in TMD. In this study twenty markers from sensory, autonomic, inflammatory, and psychological domains were evaluated. The results revealed that first overall dysregulation in multiple system domains (OR = 1.6, 95% confidence interval [CI] = 1.4–1.8), particularly in the sensory (OR = 1.9, 95% CI = 1.3–2.9) and the psychological (OR = 2.1, 95% CI = 2.1–2.7) domains, were associated with increased likelihood of being a painful TMD case and second dysregulations in individual system domains were selectively associated with the increased odds of being a TMD case with different levels of comorbid persistent pain conditions. These outcomes indicate that heterogeneous multisystem dysregulations may exist in painful TMD subgroups, and multidimensional physiological and psychological assessments can provide important information regarding pathophysiology, diagnosis, and management of pain in TMD patients.^119^

Further, based on the combination of genotypes of these SNP investigators categorize the haplotypes as low, average, and high pain sensitivity (LPS, APS and HPS). Later, investigators concluded that individuals with the LPS haplotype have higher levels of COMT enzymatic activity, which means decreased probability of developing TMD while APS or HPS haplotypes had an increased relative risk of developing TMD compared to those with “pain-resistant” LPS haplotypes.^26,120^

However, genetic association studies in TMD concluded that TMD heritability is multifaceted that mostly arises from genes encoding proteins involved in the serotonergic and catecholaminergic systems. The OPPERA study identified associations between TMD-related pain and SNPs in COMT, the serotonin receptor HTR2A, and ERA and observed that TMD-related pain, are significantly more prevalent in women than men.^117,120^ Additionally, in chronic widespread pain conditions, such as fibromyalgia, that often overlap with TMD genome-wide association studies suggest that genetic and epigenetic factors such as SLC64A4, TRPV2, MYT1L, and NRXN3 along with environmental factors play a vital role in the development of chronic pain.

There are a few studies that also have analyze the biochemical changes associated with TMD. In the study, a reduced levels of vitamin D were observed in the patients with TMD compared to control.^121^ Similarly, oxidative stress biomarkers markers 8-hydroxydeoxyguanosine and malondialdehyde (8‐OHdG) have also been analyzed. In a recent observation, higher levels of 8‐OHdG from saliva (OR: 2.68, 95% CI: 2.11–23.41) or log 8‐OHdG from serum (OR: 2.22, 95%CI: 1.71–2.88, were both related to severity of TMD pain and total antioxidant status have been found to be significantly different in saliva between TMD patients and controls.^122,123^

## Future perspectives and conclusion

Current review elucidates the potential biomarkers involved in TMD pain. Summary of Potential Biomarkers are depicted in Table 1. It is observed that by analyzing various biofluids different molecular markers can used to identify the risk factors and predicting the progression and outcomes of painful TMD conditions. In addition, the findings of changes in brain highlights the potential of neuroimaging methods as an investigating tool for understanding TMD pain conditions. Despite in the early stages of biomarker discovery, these methods along with miRNA, proteomics, genetics, and sensory markers which have added a significant knowledge to our readers would help the researchers in understanding the complexity of pain mechanism as well as impact of mood and behavior on pain processing in patients with painful TMD. It also helps the investigators to perform further studies on specific areas or selective pathways involved in TMD pain.

It is still needed to be explored whether these potential biomarkers can fulfill considerations of reliability and practicality in terms of replication of studies on heterogenous and large-scale sample size populations. In future, more clinical and experimental biomarkers research is needed on heterogenous models and patients with TMD pain and other comorbid conditions. Machine learning approaches have yielded promising results towards making predictions about states of pain in humans and animals based on neural recordings. Furthermore, discovery of new biomarkers will provide valuable insights in understanding of TMD pain pattern and develop therapeutics methods which can be integrated in the treatment of TMD.
